# Changes in healthcare utilisation during implementation of remote atrial fibrillation management: TeleCheck-AF project

**DOI:** 10.1007/s12471-023-01836-6

**Published:** 2024-01-12

**Authors:** Monika Gawałko, Konstanze Betz, Veerle Hendriks, Astrid N. L. Hermans, Rachel M. J. van der Velden, Martin Manninger, Sevasti-Maria Chaldoupi, Henk Hoogervorst, Herm Martens, Nikki A. H. A. Pluymaekers, Marieke D. Spreeuwenberg, Jeroen Hendriks, Dominik Linz

**Affiliations:** 1 Department of Cardiology, Maastricht University Medical Centre and Cardiovascular Research Institute Maastricht, Maastricht, The Netherlands; 2 1st Department of Cardiology, Medical University of Warsaw, Warsaw, Poland; 3 Institute of Pharmacology, West German Heart and Vascular Centre, University of Duisburg-Essen, Essen, Germany; 4 Department of Internal Medicine and Cardiology, Eifelklinik St. Brigida GmbH & Co. KG, Simmerath, Germany; 5 Department of Health Services Research, Care and Public Health Research Institute (CAPHRI), Faculty of Health Medicine and Life Sciences, Maastricht University, Maastricht, The Netherlands; 6 Department of Cardiology, Clinic of Medicine, Medical University of Graz, Graz, Austria; 7 Health Care Innovation and Experience Lab—Maastricht University Medical Centre+, Maastricht, The Netherlands; 8 Caring Futures Institute, College of Nursing and Health Sciences, Flinders University, Bedford Park, SA Australia; 9 Centre for Heart Rhythm Disorders, University of Adelaide and Royal Adelaide Hospital, Adelaide, SA Australia; 10 Department of Cardiology, Radboud University Medical Centre, Nijmegen, The Netherlands; 11 Department of Biomedical Sciences, Faculty of Health and Medical Sciences, University of Copenhagen, Copenhagen, Denmark

**Keywords:** Atrial fibrillation, Mobile health, Reimbursement, Healthcare utilisation

## Abstract

**Aim:**

To evaluate changes in healthcare utilisation and comprehensive packages of care activities and procedures (referred in the Netherlands to as ‘*diagnose-behandelcombinatie *(DBC) care products) during the implementation of the TeleCheck-AF approach (teleconsultation supported by app-based heart rate/rhythm monitoring) in a Dutch atrial fibrillation (AF) clinic.

**Methods and results:**

In the Maastricht University Medical Centre+ AF Clinic, data on healthcare utilisation and DBC care products for patients consulted by both a conventional approach in 2019 and the TeleCheck-AF approach in 2020 were analysed. A patient experience survey was performed. Thirty-seven patients (median age 68 years; 40% women) were analysed. With the conventional approach, 35 face-to-face consultations and 0 teleconsultations were conducted. After the implementation of TeleCheck-AF, the number of face-to-face consultations dropped by 80% (*p* < 0.001) and teleconsultations increased to 45 (*p* < 0.001). While 42 electrocardiograms (ECGs) and 25 Holter ECGs or echocardiograms were recorded when using the conventional approach, the number of ECGs decreased by 71% (*p* < 0.001) and Holter ECGs or echocardiograms by 72% (*p* < 0.001) with the TeleCheck-AF approach. The emergency department patient presentations showed no statistically significant change (*p* = 0.33). Overall, 57% of medium-weight DBC care products were changed to light-weight ones during implementation of the TeleCheck-AF approach. Patient satisfaction with the TeleCheck-AF approach was high.

**Conclusion:**

The implementation of TeleCheck-AF led to a change in healthcare utilisation, a change from medium-weight to light-weight DBC care products and a reduction in patient burden. These results created the basis for a new reimbursement code for the TeleCheck-AF approach in the Netherlands.

**Supplementary Information:**

The online version of this article (10.1007/s12471-023-01836-6) contains supplementary material, which is available to authorized users.

## What’s new?

- TeleCheck-AF is a mobile health infrastructure initiated to maintain comprehensive atrial fibrillation (AF) management.
- The digital transformation of healthcare delivery models via the TeleCheck-AF approach has the potential to improve access to remote AF management.
- The results of the present study created the basis for a new reimbursement code for the TeleCheck-AF approach in the Netherlands.
- Experience from TeleCheck-AF implementation can provide a roadmap for the future development of digital AF care reimbursement models in the Netherlands and worldwide.

## Introduction

Atrial fibrillation (AF) is the most prevalent sustained cardiac arrhythmia [1]. The prevalence of AF is estimated to be between 2 and 4%, with a 2.3-fold increase expected by the year 2060 [1]. The number of AF hospitalisations and required outpatient clinic consultations continues to rise and represents a growing cost and workload burden [2]. The ongoing digital transformation of healthcare delivery models, which is characterised by the use of telemedicine and mobile health (mHealth)-based solutions, has the potential to improve access to early, comprehensive, remote AF management [3–6].

TeleCheck-AF is a well-characterised and standardised digital care pathway, which has been developed and co-designed with patients and healthcare professionals. It consists of a structured teleconsultation (‘Tele’) preceded by an app-based, on-demand heart rate/rhythm monitoring infrastructure (‘Check’) and the consecutive integration of the app-derived findings into a comprehensive AF management system (‘AF’) [7–9]. To date, the TeleCheck-AF approach has been used for more than 6000 patients from 41 centres in 15 countries. Based on our previous results, the implementation of the TeleCheck-AF infrastructure is easy, and the project could be set up in a short time in different European centres [10]. Patients are highly adherent to and motivated by this approach [11]. However, the lack of standardised reimbursement models for such digital AF care pathways was identified as a relevant burden for the clinical implementation of TeleCheck-AF. Additionally, in a recent survey, 73.6% of respondents confirmed a lack of standardised reimbursement fees in their countries for physician consultations related to digital devices [12].

To improve Dutch reimbursement models to accelerate transformation towards telemedicine-based AF management, the Maastricht University Medical Centre (MUMC+) together with Dutch health insurers collected data on changes in healthcare utilisation and comprehensive packages of care activities and procedures for patients’ diagnosis and treatment, which in the Netherlands are referred to as ‘*diagnose-behandelcombinatie* (DBC) care products’ (see Methods) during the implementation of the TeleCheck-AF approach in the MUMC+ AF Clinic. Herein, we report those changes and summarise the insights gained from TeleCheck-AF in a roadmap for future mHealth reimbursement strategies.

## Methods

### TeleCheck-AF

A detailed description of the TeleCheck-AF infrastructure is provided elsewhere [9]. Briefly, patients are provided with an mHealth prescription to use a *Conformité Européenne*-marked and Food and Drug Administration-approved, photoplethysmography (PPG)-based heart rate/rhythm monitoring app (FibriCheck), which has been validated previously [13]. Patients collect heart rate/rhythm recordings 3 times a day and, in case of symptoms, during 1 week prior to a scheduled teleconsultation. Moreover, patients receive educational information about AF, its complications and treatment via the app. Before the teleconsultation, treating physicians or AF nurses have access to a secure cloud to evaluate measurements. During the teleconsultation, detailed history taking and adaptation of the treatment plan, if indicated, are performed.

### DBC care products

Reimbursement of healthcare provided to patients in the Netherlands is based on DBC care products, comprehensive packages that encompass all care activities and procedures required, on average, to ascertain the diagnosis and perform the required treatment of a patient. The DBC care product also contains information on the reimbursement rates for healthcare practitioners as well as reimbursement of the healthcare service provided. Further elaboration on the DBC care product set-up model can be found in Fig. S1 (Electronic Supplementary Material). To minimise the influence of yearly changing rates of reimbursement for each DBC care product, we standardised the reimbursement per DBC care product, using publicly available data for 2020 from the Dutch healthcare authority (https://www.opendisdata.nl).

### Inclusion criteria

Patients with AF who were followed up by both a conventional approach (end of DBC care product in January-December 2019) and by the TeleCheck-AF approach (end of DBC care product in April-August 2020) in the MUMC+ AF Clinic were included in this prospective case-crossover analysis. Patients were further included if their follow-up appointment at the AF Clinic in 2020 had already been scheduled during an AF Clinic appointment in 2019.

### Exclusion criteria

Patients with AF without a scheduled follow-up appointment in 2020, with DBC care product codes related to AF ablation, pacemaker implantations and other invasive procedures, were not included in order to diminish bias in the reimbursement calculation.

### Data collection

We used only one 3‑month DBC care product period from 2019, which was compared with a 3-month DBC care product period from 2020 for the TeleCheck-AF approach. Four different DBC care product codes were included in the analysis and are summarised in Table S1 (Electronic Supplementary Material) with related reimbursement. In the analysis, healthcare utilisation during a DBC care product period in terms of numbers and modes of outpatient contacts (teleconsultation vs face-to-face), the number of emergency department presentations and the use of diagnostic tests, i.e. electrocardiogram (ECG), Holter monitoring, echocardiogram, as well as the weighting of the DBC care products were compared between the conventional approach in 2019 and the TeleCheck-AF approach in 2020.

### Patient experience

Patients received an experience survey including questions about using the app and being treated with the TeleCheck-AF approach. The complete questionnaire is described in detail elsewhere [10].

### Statistical analysis

All continuous variables were assessed for normality by the Shapiro-Wilk test. Continuous variables were presented as median (interquartile range) or mean (to show the ratios of number of consultations/diagnostic tests per number of patients). Categorical variables were presented as numbers (*n*) with percentages (%). Differences in continuous parameters were compared using the non-parametric Kruskal-Wallis test (three-group comparison) or parametric independent-sample *t*-test (two-group comparison), as applicable. For the comparison of categorical data, Pearson’s chi-square test was used. A two-sided *p *value of < 0.05 was considered statistically significant. For database management and statistical analysis, we used SAS 14.1 (SAS Institute Inc., Cary, NC, USA).

## Results

**Fig. 1:**
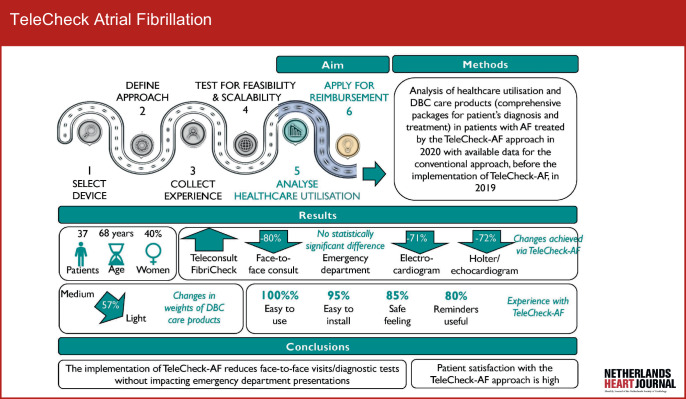
Infographic: Summary of our study including aims, methods, results and conclusions as well as a brief roadmap of mobile health device implementation in the healthcare system

Thirty-seven patients (median age 68 (58–73) years; 40% women) with available data on healthcare utilisation and DBC care products, who were consulted by both the conventional approach in 2019 and TeleCheck-AF approach in 2020, were analysed. Detailed characteristics of the study population are provided in Table S2 (Electronic Supplementary Material). An infographic summarising our study (aims, methods, results and conclusions) as well as a roadmap for implementation of mHealth are presented in Fig. 1.

### Changes in healthcare utilisation

**Fig. 2:**
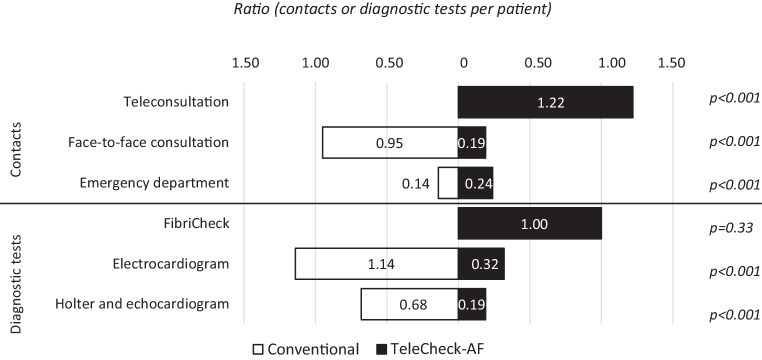
Comparison of the number of contacts/diagnostic tests between conventional and TeleCheck-AF approach

Using the conventional approach (before the implementation of TeleCheck-AF) there were 35 face-to-face consultations and 0 teleconsultations. With the TeleCheck-AF approach, there were 7 face-to-face consultations (decrease of 80%, *p* < 0.001) and 45 teleconsultations (*p* < 0.001). While 42 ECGs and 25 Holter ECGs/echocardiograms were recorded when using the conventional approach, the number of ECGs with the TeleCheck-AF approach was 12 (decrease of 71%, *p* < 0.001) and the number of Holter ECGs/echocardiograms was 7 (decrease of 72%, *p* < 0.001). Importantly, the number of patient presentations at the emergency department did not differ statistically significantly between the conventional and the TeleCheck-AF approach (5 vs 9 visits, respectively; *p* = 0.33; Fig. 2). However, the low number of emergency department visits might have impacted the statistical significance.

As we used only one DBC care product from 2019 that was compared with one DBC care product from 2020 for the TeleCheck-AF approach, we analysed additionally whether this DBC care product from 2019 was representative of all the DBC care products ending in 2019. Indeed, as seen in Figure S2 (Electronic Supplementary Material), the number of contacts and diagnostic tests was comparable between one DBC care product from 2019 (that compared with one DBC care product from 2020) and all DBC care products ending in 2019.

On average, the duration of a face-to-face consultation was 20 min. The evaluation of FibriCheck records and the teleconsultation, as part of the TeleCheck-AF approach, lasted 2.0 (0.8–2.8) min and 3.0 (0.5–6.0) min, respectively. This resulted in a 75% reduction in the length of the consultation when transitioning from face-to-face consultations to teleconsultations. Implementation of the TeleCheck-AF approach reduced expenditure related to travel time for a face-to-face consultation, emergency department presentations as well as hospital visits for diagnostic tests (which were not part of face-to-face consultations or emergency department visits), statistically significantly (by 68%; *p* < 0.001).

### Integration of TeleCheck-AF

According to reports of the treating physicians, in 4 (11%) patients rate/rhythm control medications were adjusted, 6 (16%) patients were scheduled for an electrical cardioversion and 1 (2.7%) patient had changes in rate control medication and was scheduled for pulmonary vein isolation (Fig. S3A, Electronic Supplementary Material). In 59% of patients, a teleconsultation was scheduled as a follow-up appointment, and in 32% of patients FibriCheck was requested again (Fig. S3B, Electronic Supplementary Material), mostly in patients in whom AF was detected during the initial teleconsultation.

### Patient experience

According to replies from the patient experience survey regarding the TeleCheck-AF infrastructure, patients agreed that the app was easy to use (100%), easy to install (95%) and that they would like to use the app in the future (55%). The PPG-based heart rate and rhythm recording gave patients a feeling of safety (85%) and automated reminders were useful (80%) (Fig. S4, Electronic Supplementary Material).

### Impact on DBC care products

**Fig. 3:**
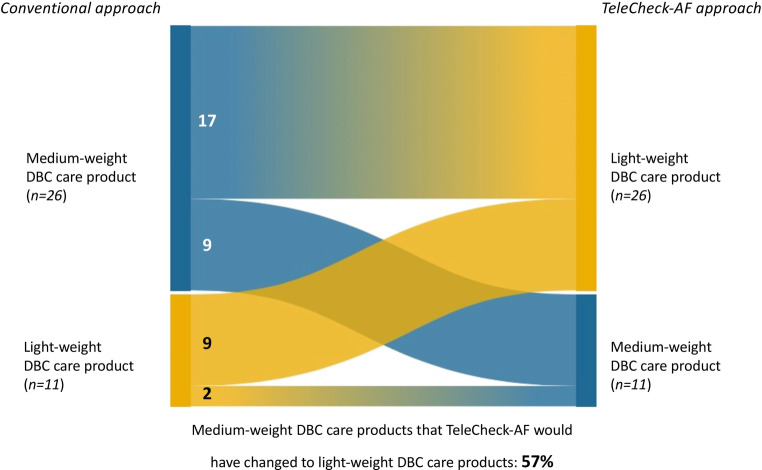
Changes in type of diagnosis-treatment combination (*DBC*) care product from conventional to TeleCheck-AF approach

Overall, 57% of medium-weight DBC care products were changed to light-weight DBC care products during implementation of the TeleCheck-AF approach (Fig. 3). This resulted in a 29% lower reimbursement per patient in the TeleCheck-AF approach in 2020 as compared to the conventional approach in 2019 (Fig. S5, Electronic Supplementary Material). Higher prevalence of previous thromboembolic events was the only difference in baseline characteristics between the group of patients in whom reimbursement was increased (37%) versus the group of patients with no change/decreased reimbursement when comparing 2020 with 2019 (5.6%, *p* = 0.04) (Table S2, Electronic Supplementary Material).

In the Netherlands, Dutch health insurers reimbursed approximately € 49,601,505 for medium-weight and light-weight DBC care products for AF outpatient treatment in 2020. Extrapolating our study results on a national level, the assumed shift from medium-weight to light-weight DBC care products could potentially decrease the amount reimbursed by Dutch health insurers for DBC care products for AF outpatient treatment by € 11,200,400 to € 38,401,105 (−23%).

## Discussion

TeleCheck-AF is a well-defined and standardised mHealth-based care pathway for the remote management of patients with AF in outpatient clinics, known for its effective scalability and for being patient-friendly, as previously described [8–11]. Implementation of the TeleCheck-AF approach in the MUMC+ AF Clinic resulted in a significant reduction in healthcare utilisation (face-to-face visits and diagnostic tests), leading to a change in DBC care products. Specifically, there was a decrease in medium-weight and an increase in light-weight DBC care products compared to usual care in 2019, resulting in a disproportionate drop in reimbursement received by healthcare providers, as the actual costs for the TeleCheck-AF approach, e.g. for technology, personnel and implementation procedures, are not covered. This highlights the inflexibility of reimbursement codes, which have not been adapted to the ongoing digital transformation of healthcare delivery models. This problem has previously been noted in surveys to represent a significant burden for future mHealth implementation [3, 12]. Based on the healthcare utilisation and reimbursement data presented in this paper, as well as previously published data on feasibility [8–11], the MUMC+, together with a major Dutch health insurance company, requested an optional billing code from the Dutch Healthcare Authority (NZa). This optional billing code (‘*Telecheck atriumfibrilleren*’; TB/REG-21679-01, https://puc.overheid.nl/nza/doc/PUC_695550_22/1/) is specifically for use in the Netherlands. The code is not for the direct reimbursement of costs involved in the use of mHealth, but can be used to compensate for the financial gap resulting from an overproportional drop in reimbursement for AF disease management through use of the TeleCheck-AF approach. Based on the experience and data that will be collected 1 year after the introduction of the optional billing code, it will be discussed whether the TeleCheck-AF approach should be integrated in the existing DBC care product system and whether this on-demand mHealth approach can be expanded to other clinical scenarios.

Some specific limitations should be considered when PPG is used in the TeleCheck-AF setting. Compared to ECG, PPG measurements are not able to definitely diagnose underlying heart rhythms and cannot differentiate between ventricular and supraventricular beats or sinus tachycardia and atrial flutter [14, 15]. Nevertheless, these limitations can be addressed by confirming PPG-detected arrhythmia episodes by ECG documentation [15]. Additionally, the quality of PPG signals can be impacted by various factors, such as the effects of ambient light, accommodating different skin pigmentations and conditions (tattoos, low skin temperature, eczema, etc.), or movement artefacts [14]. Moreover, irrespective of the technology used, intermittent recordings (e.g. 3 measurements a day as used in TeleCheck-AF) may miss asymptomatic and short arrhythmia episodes, which could otherwise be captured by continuous monitoring. In our study, we present data from a patient survey, suggesting that continuous ECG monitoring (e.g. Holter) may be burdensome for patients, whereas intermittent PPG recordings (3 times a day) were accepted as a patient-friendly alternative.

By implementing TeleCheck-AF as a telemedicine approach, the potential burden on patients caused by, for example, travel costs and time investment could be significantly lowered. Additionally, it may represent a good strategy to prepare for future crises, when attendance at outpatient clinics or travelling to the hospital is not possible or undesirable. Integrated AF management through the TeleCheck-AF infrastructure could therefore be an example of creating alternatives to standard care for sustainable and future-oriented telemedical care pathways. However, data from randomised controlled trials and cost-effectiveness analysis are needed to support these findings.

**Fig. 4:**
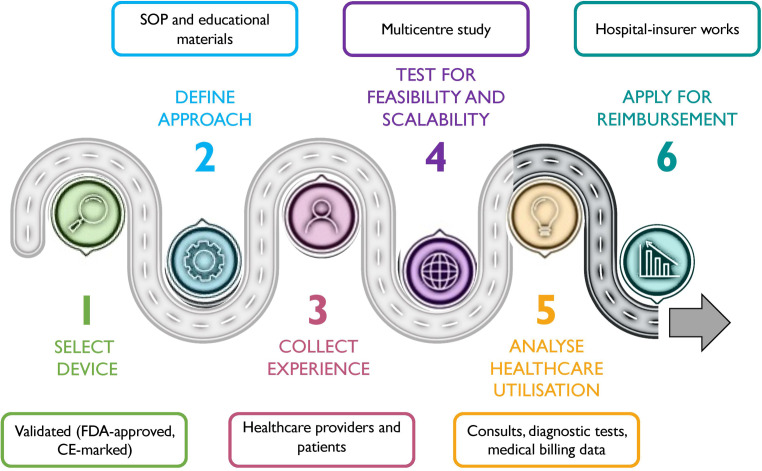
Roadmap of mobile health device implementation in healthcare system. *CE* Conformité Européenne, *FDA* Food and Drug Administration, *SOP* standard operating procedure. See also description in Discussion section

The development of the TeleCheck-AF project and a reimbursement model for this specific clinical scenario involved a multidisciplinary team consisting of cardiologists, electrophysiologists, nurses and patients, as well as regulatory agencies, health insurance companies, hospital finance departments and mHealth companies. The FibriCheck app was selected for the TeleCheck-AF approach [13, 16] after being validated prior to creation of the AF management pathway [17] (step 1). To define the TeleCheck-AF approach, standard operating procedure documents [9], involving a case manager and patient education [8], were prepared. Also, an educational, structured, stepwise practical guide on how to interpret PPG signals was developed for healthcare professionals to facilitate the use of an on-demand PPG technology in a clinical scenario [14] (step 2). Extensive feedback from healthcare providers using the TeleCheck-AF approach and patient experience were collected and used to continuously refine the approach to overcome challenges related to mHealth-guided pathways [7] (step 3). With expansion of the project, the feasibility and scalability of TeleCheck-AF were proven [10]. Centre and patient characteristics as well as data on patient motivation and adherence to such an approach were collected [11], and the ability to integrate mHealth data in clinical decision-making processes was demonstrated [18] (step 4). Data presented in this article summarise the results of activities focusing on steps 5 and 6*.* Together with the finance department at MUMC+, a major Dutch health insurance company and the Dutch Healthcare Authority (NZa), data on healthcare utilisation, DBC care products, safety and effectiveness were collected (step 5), which formed the basis for discussions on reimbursement (step 6). The herein proposed roadmap (Fig. 4) for future mHealth implementation can support the ongoing digital transformation of healthcare delivery models and the design of further reimbursement strategies for broader and sustained mHealth use.

### Limitations

TeleCheck-AF was initiated during the COVID-19 pandemic, which may have impacted healthcare utilisation and admission rates due to limited availability and regional restrictions. To address this limitation, we included only patients with a routine follow-up visit at the MUMC+ AF Clinic in 2019, for whom a routine AF clinic follow-up appointment was already scheduled for 2020. We excluded patients with a first consultation and patients with DBC care product codes related to AF ablation, pacemaker implantations and other invasive procedures. To validate the impact of the TeleCheck-AF approach on long-term outcomes and cost-effectiveness in AF populations beyond the COVID-19 pandemic, evidence from a randomised clinical trial is needed.

## Conclusions

The implementation of TeleCheck-AF was associated with a change in health care utilisation and a disproportional decrease in reimbursement by health insurers due to a change from medium-weight to light-weight DBC care products. Its implementation decreased the potential burden on patients caused by, for example, travel costs and time investment. These results were used as the basis for the development of a new optional reimbursement code for the TeleCheck-AF approach in the Netherlands. This example can serve as a roadmap for future development of digital AF care reimbursement models in the Netherlands and worldwide. Multicentre prospective studies with longer follow-up periods are necessary to determine the long-term impact of TeleCheck-AF on healthcare utilisation, safety, efficacy and costs involved in the care of patients with AF.

### Supplementary Information

Supplemental Figure S1. Diagnosis-treatment combination (DBC) set up model. Supplemental Figure S2. Comparison of contacts/ diagnostic tests between diagnosis-treatment combination (DBC) ending in 2019 with DBC with TeleCheck-AF approach in 2020 and all DBC ending in 2019. Supplemental Figure S3. Integration of FibriCheck results in the clinical decision-making process within the TeleCheck-AF approach Supplemental Figure S4. Patients’ experience with TeleCheck-AF approach (*n* = 20). Supplemental Figure S5. Comparison between reimbursement the consultations/ diagnostic tests in conventional and TeleCheck-AF approach. Supplemental Table S1. Reimbursement of selected diagnosis-treatment combination (DBC) care products. Data from https://www.opendisdata.nl. Supplemental Table S2. Baseline characteristics of study population.
