# Supplementary material for: Changes in healthcare utilisation during implementation of remote atrial fibrillation management: TeleCheck-AF project
Source: Neth Heart J. 2024 Jan 12;32(3):130–9. doi: 10.1007/s12471-023-01836-6 (PMC10884376; doi:10.1007/s12471-023-01836-6)
Supplement: Supplementary file 1 — Supplemental Figure S1. Diagnosis-treatment combination (DBC) set up model. Supplemental Figure S2. Comparison of contacts/ diagnostic tests between diagnosis-treatment combination (DBC) ending in 2019 with DBC with TeleCheck-AF approach in 2020 and all DBC ending in 2019. Supplemental Figure S3. Integration of FibriCheck results in the clinical decision-making process within the TeleCheck-AF approach Supplemental Figure S4. Patients’ experience with TeleCheck-AF approach (n = 20). Supplemental Figure S5. Comparison between reimbursement the consultations/ diagnostic tests in conventional and TeleCheck-AF approach. Supplemental Table S1. Reimbursement of selected diagnosis-treatment combination (DBC) care products. Data from https://www.opendisdata.nl. Supplemental Table S2. Baseline characteristics of study population. [file 12471_2023_1836_MOESM1_ESM.pdf]

Supplemental material online

Changes in healthcare utilisation during implementation of remote atrial fibrillation management: TeleCheck-AF project

Monika Gawałko[1,2,3]†, Konstanze Betz[1,4]†, Veerle Hendriks[5], Astrid NL Hermans[1], Rachel MJ van der Velden[1], Martin Manninger[6], Sevasti-Maria Chaldoupi[1], Henk Hoogervorst[1], Herm Martens[7], Nikki AHA Pluymaekers[1], Marieke D Spreeuwenberg[5], Jeroen Hendriks[8,9], Dominik Linz[1,10,11]

[1] Department of Cardiology, Maastricht University Medical Centre and Cardiovascular Research Institute Maastricht, Universiteitssingel 50, 6229 ER Maastricht, the Netherlands.

[2] 1st Department of Cardiology, Medical University of Warsaw, Banacha 1A, 02-197 Warsaw, Poland.

[3] Institute of Pharmacology, West German Heart and Vascular Center, University Duisburg-Essen, Hufelandstraße 55, Essen 45147, Germany.

[4] Department of Internal Medicine and Cardiology, Eifelklinik St. Brigida GmbH & Co. KG, Kammerbruchstrasse 8, 52152 Simmerrath, Germany

[5] Department of Health Services Research, Care and Public Health Research Institute (CAPHRI), Faculty of Health Medicine and Life Sciences, Maastricht University, Duboisdomein 30, Maastricht 6229 GT, The Netherlands.

[6] Department of Cardiology, Clinic of Medicine, Medical University of Graz, Auenbruggerplatz 15, 8036 Graz, Austria

[7] Health Care Innovation and Experience Lab – Maastricht University Medical Centre+, P. Debeyeplein 25, 6202 AZ, Maastricht, the Netherlands.

[8] Caring Futures Institute, College of Nursing and Health Sciences, Flinders University, Sturt Road, Bedford Park, Adelaide, SA 5042, Australia.

[9] Centre for Heart Rhythm Disorders, University of Adelaide and Royal Adelaide Hospital, North Terrace, Adelaide, SA 5000, Australia.

[10] Department of Cardiology, Radboud University Medical Centre, Geert Grooteplein 28, Nijmegen 6525 GA, The Netherlands.

[11] Department of Biomedical Sciences, Faculty of Health and Medical Sciences, University of Copenhagen, Blegdamsvej 3B, Copenhagen 2200, Denmark.

†shared first authorship

***Address for Correspondence**

Dominik Linz, MD, PhD

Department of Cardiology, Maastricht University Medical Centre and Cardiovascular Research Institute Maastricht, Universiteitssingel 50, 6229 ER Maastricht, the Netherlands.

E dominik.linz@mumc.nl | T +31(0)43-3875093 | M +31(0)6-123 99 182

Supplemental Figure S1. Diagnosis-treatment combination (DBC) set up model.

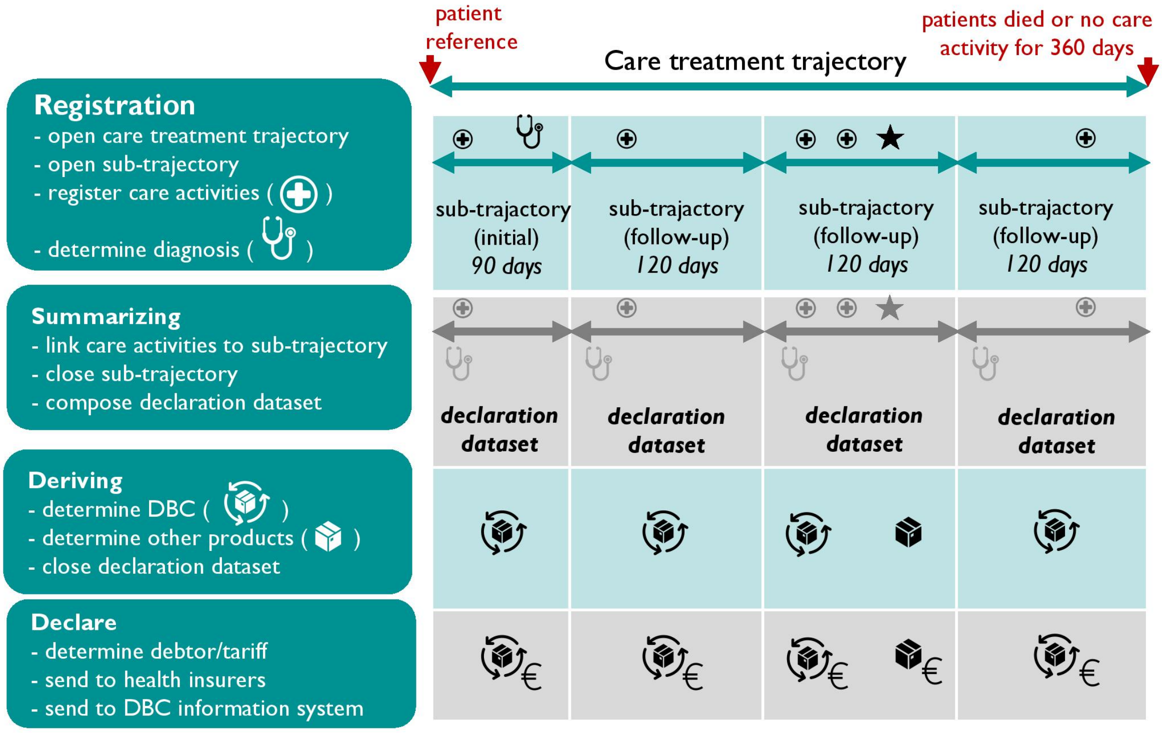

Legend: *Medical billing of care treatment trajectory in the Netherlands.*
The DBC care product is set up based on the four-step model:

1. Registration. *Once a patient visits the hospital for a specific complaint, a care treatment trajectory is opened. Opening a care treatment trajectory automatically opens a sub-trajectory. A sub-trajectory is a defined period within the care treatment trajectory for which the care provided is invoiced, marked by the cut-off moments. The care treatment trajectory contains one or more sub-trajectories. An initial sub-trajectory has a maximum duration of 90 days. If the care activity has not been completed, a new sub-trajectory can be opened after the initial sub-trajectory for a maximum period of 120 days. The care treatment trajectory is closed if no care activities are registered or planned in the future for a period of three times 120 days after the conclusion of a sub-trajectory, or immediately after the death of the patient.*

In this registration process, the healthcare provider gradually records which care activities have been carried out to establish a diagnosis and to treat a complaint or condition per sub-trajectory.

2. Summary. *The registered information (diagnosis and care activities) is summarized per sub-trajectory in one structured dataset.*

3. Derivation. *After the sub-trajectory has been completed, the care provider sends data about the contributed care to a grouper (computer application). A grouper derives the DBC care product based on the data supplied (the claim data set).*

4. Declaration. *The DBC care product that is derived by the grouper is given a declaration code. Healthcare providers can charge healthcare based on this declaration code. In general, there are three weights for DBC care products: light (≤ € 200), medium (€ 300-500) and heavy (≥ € 600). In the cardiology outpatient clinic either light or medium weight DBC are usual. A light weight DBC care product is 1-2 outpatient clinic visits (including remote consultations) with or without electrocardiogram (ECG). As soon as a patient has an additional examination, e.g., Holter or an echocardiogram examination, or additional visit (≥3) with or without ECG, the weight of the DBC care product increases and turns into a medium weight DBC care product. If there is both an echocardiogram and a Holter performed, only one of the diagnostic tests adds to the weight of the DBC care product, and if there are three echocardiogram examinations within one DBC care product only the first one adds to the weight. Noteworthy, it is possible that two patients who seem to have the same DBC care*

product with the same activities have different reimbursement, as reimbursement may vary per every quartile (3 months). To minimize the influence of changing reimbursement between each DBC care product, we standardized the reimbursement per DBC care product, using reimbursement from 2020 from publicly available data on the DBC care product information system from the Dutch healthcare authority (https://www.opendisdata.nl).

Supplemental Figure S2. Comparison of contacts/ diagnostic tests between diagnosis-treatment combination (DBC) ending in 2019 with DBC with TeleCheck-AF approach in 2020 and all DBC ending in 2019.

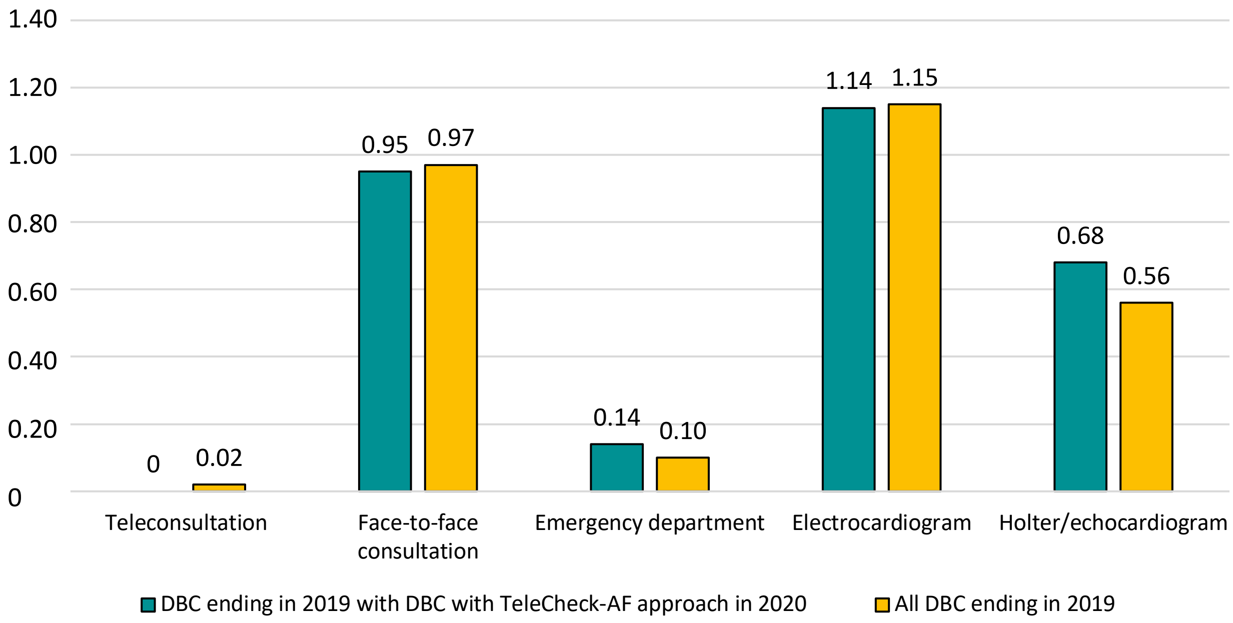

Abbreviations: *DBC, diagnosis-treatment combination*

Supplemental Figure S3. Integration of FibriCheck results in the clinical decision-making process within the TeleCheck-AF approach.

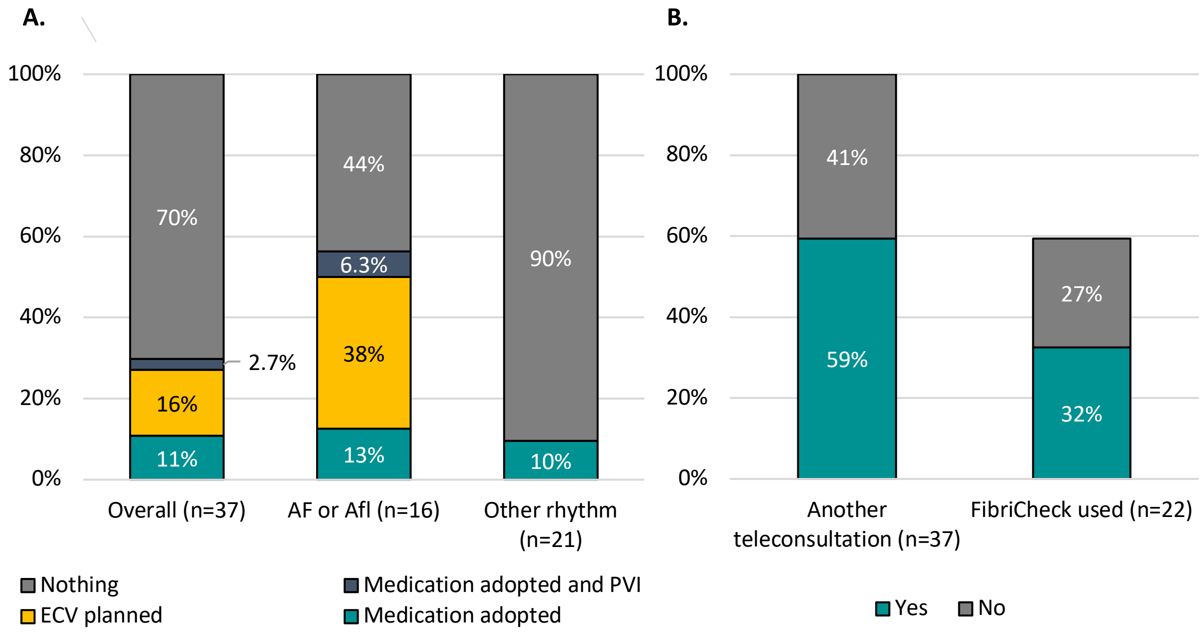

Abbreviations: AF, atrial fibrillation; AFl, atrial flutter; ECV, electrical cardioversion; PVI, pulmonary vein isolation

Legend: Plot A shows proportion of patients (n=37) with ECV planned, medication adopted with or without PVI or no changes in treatment after first FibriCheck usage. Data are also provided regarding the detected rhythm during the first 7-day FibriCheck usage: AF or AFl (n=16) or other rhythm (n=21). Among 16 patients with AF or AFl, there was suspicion of AFl in 2 patients because of absence of respiratory arrhythmia, a strict heart rate and presence of blocked beats in the 1 min photoplethysmography recordings, which was confirmed as typical AFl by electrocardiogram.

Plot B shows the additional teleconsultations needed after the first teleconsultation (n=22) and proportion of additional FibriCheck usage needed before additional teleconsultation (n=12).

Note: The figures may not add up due to rounding.

Supplemental Figure S4. Patients' experience with TeleCheck-AF approach (n=20).

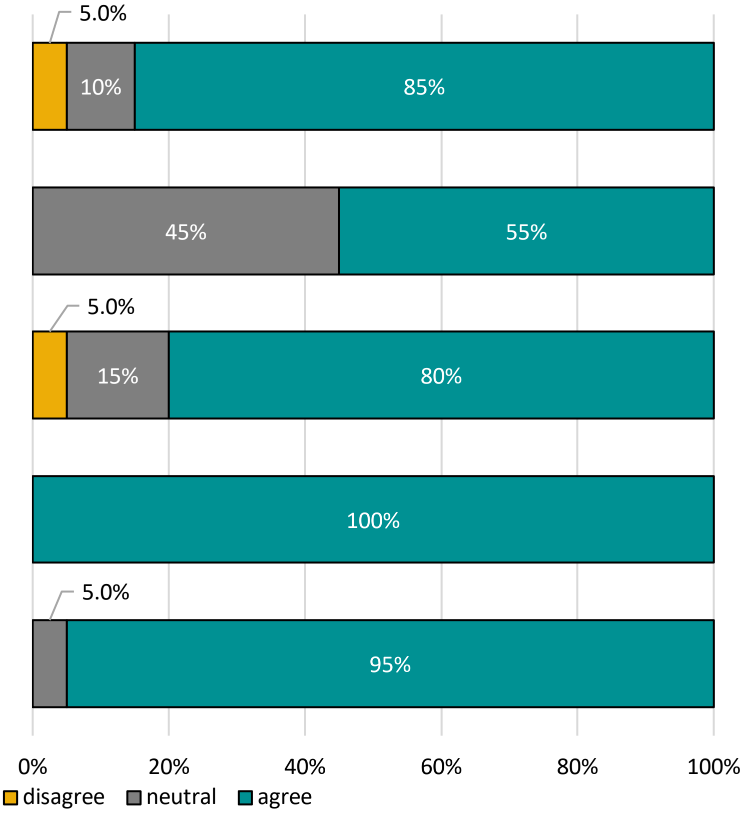

Supplemental Figure S5. Comparison between reimbursement the consultations/ diagnostic tests in conventional and TeleCheck-AF approach.

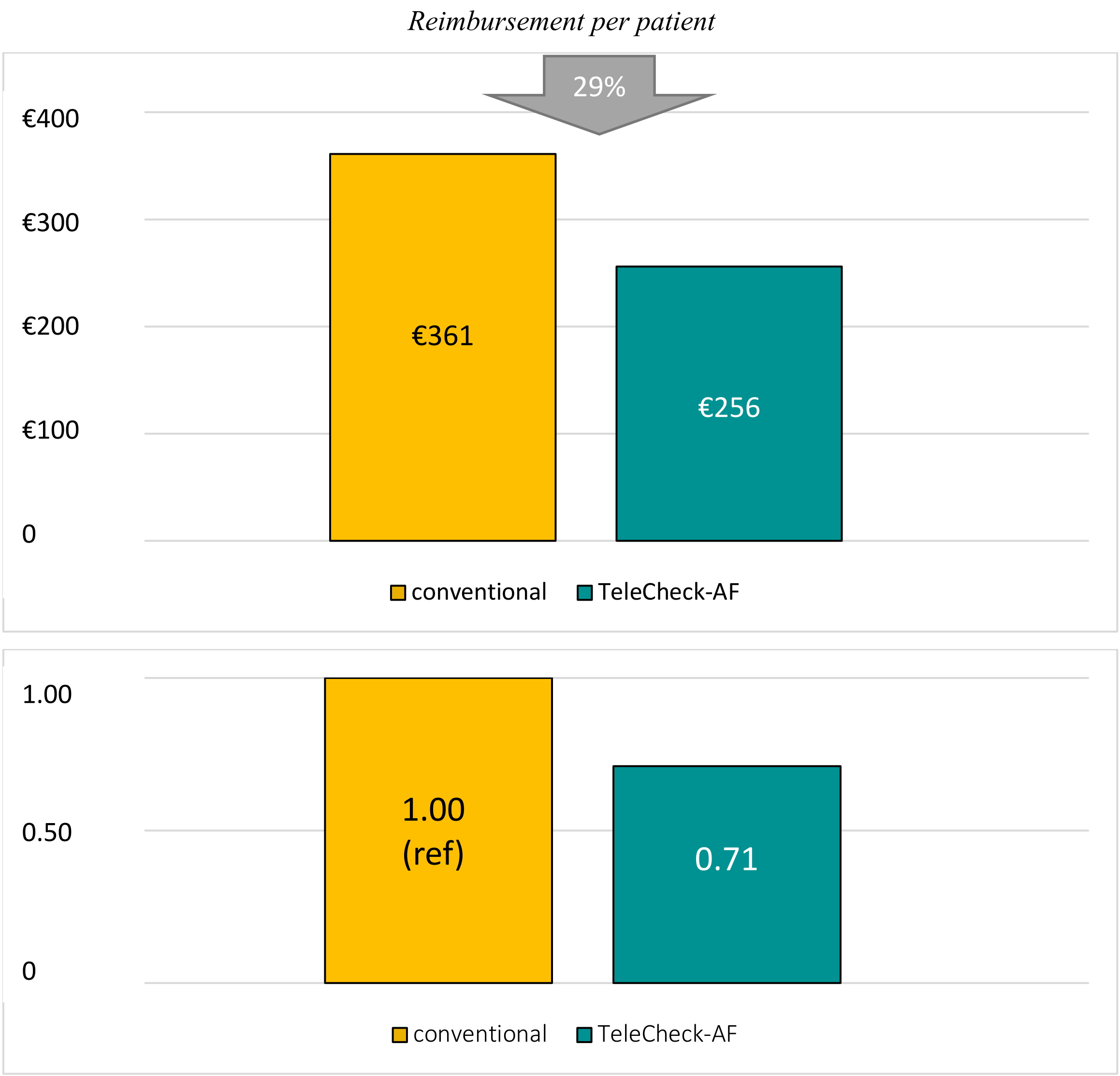

Supplemental Table S1. Reimbursement of selected diagnosis-treatment combination (DBC) care products. Data from https://www.opendisdata.nl.

DBC care product	Weight	Code	Description	Reimbur-sement
SVT/AF/AFl intervention	Light	099899072	1 or 2 outpatient clinic visits/remote consultations in cardiology in the event of a cardiac arrhythmia	€ 185
SVT/AF/AFl follow-up	Light	219699019	1 or 2 outpatient clinic visits / remote consultations after heart surgery or angioplasty	€ 170
SVT/AF/AFl intervention	Medium	099899063	Diagnostics/surgery and/or more than 2 outpatient clinic visits/remote consultations in cardiology in the event of a cardiac arrhythmia	€ 450
SVT/AF/AFl follow-up	Medium	219699008	Diagnostics/surgery and/or more than 2 outpatient clinic visits/remote consultations after heart surgery or angioplasty	€ 400

Abbreviations: *AF, atrial fibrillation; AFl, atrial flutter; DBC, diagnosis-treatment combination; SVT, supraventricular arrhythmia*

Supplemental Table S2. Baseline characteristics of study population.

**Baseline characteristics**	**Patients (n=37)**	**Reimbursement in TeleCheck-AF (vs conventional) approach**		
		**Not changed/ decreased (n=18)**	**Increased (n=19)**	**P value**
Age, years	68 [58-73]	69 [62-72]	67 [57-75]	0.66
Females	15 (40%)	7 (39%)	8 (42%)	1.00
Body mass index, kg/m^2	29 [27-32]	28 [27-32]	29 [26-32]	0.59
Paroxysmal atrial fibrillation<br>Persistent atrial fibrillation<br>Permament atrial fibrillation	16 (43%)<br>19 (51%)<br>2 (5.4%)	10 (56%)<br>7 (39%)<br>1 (5.6%)	6 (32%)<br>12 (63%)<br>1 (5.3%)	0.32
Previous electrical cardioversion	23 (62%)	12 (67%)	11 (58%)	0.74
Previous pharmacological cardioversion	5 (14%)	2 (11%)	3 (16%)	1.00
Previous ablation	23 (62%)	10 (56%)	13 (68%)	0.51
Rhythm control<br>Rate control	34 (92%)<br>3 (8.1%)	17 (94%)<br>1 (5.6%)	17 (89%)<br>2 (11%)	1.00
Heart failure	4 (11%)	2 (11%)	2 (11%)	1.00
Vascular disease	4 (11%)	1 (5.6%)	7 (37%)	0.042
Previous thromboembolic events	8 (22%)	4 (22%)	0 (0%)	0.05
Hypertension	19 (51%)	7 (39%)	12 (63%)	0.19
Diabetes mellitus	5 (14%)	2 (11%)	3 (16%)	1.00
CHA_2DS_2-VASc score	2.0 [1.0-4.0]	2.0 [1.0-4.0]	3.0 [1.0-4.0]	0.23
Medications				
Oral anticoagulation	34 (92%)	16 (89%)	18 (95%)	0.60
Renin angiotensin aldosterone system antagonists	14 (38%)	7 (39%)	7 (37%)	1.00
Beta-blockers	19 (51%)	9 (50%)	10 (53%)	1.00
Digoxin	6 (16%)	2 (11%)	4 (21%)	0.66
Antiarrhythmic drugs	6 (16%)	2 (11%)	4 (21%)	0.66
Calcium channel blockers (non/dihydropiridine)	12 (32%)	8 (44%)	4 (21%)	0.17
Diuretics	11 (30%)	4 (22%)	7 (37%)	0.48
